# CaMKII regulation of cardiac K channels

**DOI:** 10.3389/fphar.2014.00020

**Published:** 2014-02-21

**Authors:** Julian Mustroph, Lars S. Maier, Stefan Wagner

**Affiliations:** Department of Cardiology, University Medical Center GöttingenGöttingen, Germany

**Keywords:** CaMKII, K channel, heart failure, action potential, arrhythmias

## Abstract

Cardiac K channels are critical determinants of cardiac excitability. In hypertrophied and failing myocardium, alterations in the expression and activity of voltage-gated K channels are frequently observed and contribute to the increased propensity for life-threatening arrhythmias. Thus, understanding the mechanisms of disturbed K channel regulation in heart failure (HF) is of critical importance. Amongst others, Ca/calmodulin-dependent protein kinase II (CaMKII) has been identified as an important regulator of K channel activity. In human HF but also various animal models, increased CaMKII expression and activity has been linked to deteriorated contractile function and arrhythmias. This review will discuss the current knowledge about CaMKII regulation of several K channels, its influence on action potential properties, dispersion of repolarization, and arrhythmias with special focus on HF.

## INTRODUCTION

**FIGURE 1:**
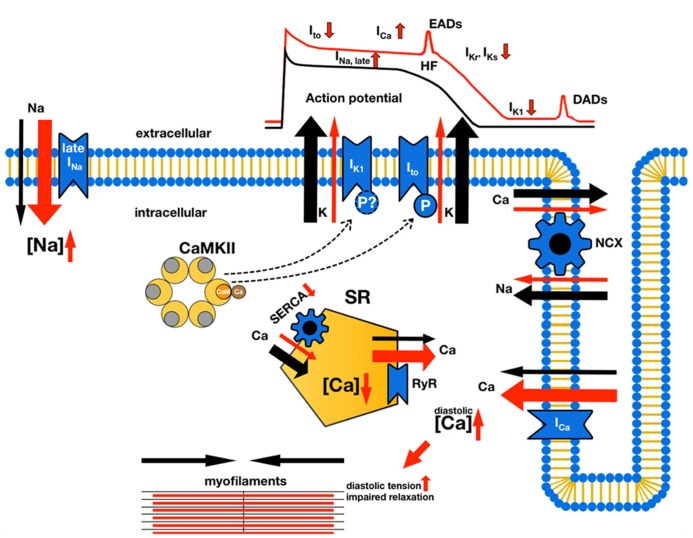
**Electro-mechanical remodeling in ventricular myocytes in HF.** Normal currents are indicated by black arrows, while changes in HF are indicated by red arrows and changes in size to indicate an increase or decrease in current density. CaMKII effects on potassium currents are indicated by bar-headed lines (in this figure only CaMKII effects on K currents are shown, for a detailed review of CaMKII effects refer to Maier and Bers, 2007). Decreased expression and function of repolarizing K currents (I_to_, I_Kr_, I_Ks_, I_K1_), for instance due CaMKII-mediated effects leads to prolongation of the AP duration. This can result in greater systolic Ca entry through voltage-gated Ca channels, but also Na entry through increased late Na current (via voltage-gated Na channels, Shryock et al., 2013). Increased cytosolic Na concentrations, a feature also observed in HF (Pieske et al., 2002), together with prolonged AP duration also impairs the Ca export function of cardiac Na/Ca exchanger (Bers, 2002a), which further aggravates the net gain in cytosolic Ca. In the face of a reduced function of the sarcoplasmic reticulum (SR) Ca ATPase in HF **(SERCA;** [Bibr B30]), this Ca remains in the cytosol thereby dramatically impairing diastolic function. Moreover, increased depolarizing currents (Na and Ca currents) during the plateau phase of the AP could lead to early afterdepolarizations (EADs), while increased diastolic SR Ca leak through ryanodine-receptor 2 (RyR) facilitates delayed afterdepolarizations (DADs).

Heart failure (HF) is a leading cause of death in western countries (United States and Europe), (Neumann et al., 2009; Go et al., 2012; Nichols et al., 2013) but also in developing countries like China (Hu et al., 2012). Morbidity in HF is characterized by contractile dysfunction and an increased propensity for arrhythmias (Luo and Anderson, 2013). Both are known consequences of the electro-mechanical remodeling of the cardiomyocyte. It is well established that reduced expression of K channels in hypertrophied and failing myocardium (Kääb et al., 1996) can lead to action potential (AP) prolongation, which is known to be pro-arrhythmogenic. Moreover, AP prolongation also leads to greater systolic Ca entry through voltage-gated L-type Ca channels (Ca_V_1.2) and impairs the Ca export function of cardiac Na/Ca exchange (NCX, Bers, 2002a), which results in cytosolic Ca overload and dramatically impairs diastolic contractile function (**Figure 1**).

Thus, understanding the mechanisms that are involved in the regulation of cardiac K channel expression and function in HF could greatly improve patient treatment.

Ca/calmodulin-dependent protein kinase II (CaMKII) has been identified as an important regulator of ion channels and transporters involved in cardiac excitation–contraction coupling under physiological but also pathophysiological conditions (Maier and Bers, 2007). Increased CaMKII expression and function was found in HF and is linked to contractile dysfunction and arrhythmias. Interestingly, there is substantial evidence that CaMKII is also involved in K channel regulation (Nerbonne, 2011). This review will discuss CaMKII-dependent regulation of several cardiac potassium channels and its significance for arrhythmogenesis and contractile function in HF.

## K CHANNELS ARE IMPORTANT REGULATORS OF CARDIAC EXCITABILITY

The cardiac AP is initiated by activation of voltage-gated Na channels (Na_V_1.5). The resulting Na current (I_Na_) leads to a rapid depolarization, i.e., the AP upstroke (phase 0; Bers, 2002b). The upstroke is limited by inactivation of I_Na_ and voltage-dependent activation of transient outward K channels (K_V_4.2, K_V_4.3, and K_V_1.4 generating I_to_). I_to_ activation results in an early repolarization (notch, phase 1), thus setting the voltage plateau of the AP. Activation of L-type Ca channels generates a depolarizing Ca current (I_Ca_) that stabilizes the membrane potential during the plateau phase (phase 2). Repolarization in phase 3 is mainly caused by activation of delayed rectifying K channels [hERG (*KCNH2*)*, *Kv7.1**(*KCNQ1*), and Kv1.5 (*KCNA5)* responsible for I_Kr_, I_Ks_, and I_Kur_, respectively]. Additionally, activation of inward rectifying K channels (Kir2.x, generating I_K1_) contributes to late phase repolarization. The resting membrane potential (phase 4) is stabilized by I_K1_, but ion conductance in phase 4 is also influenced by the Na/K-ATPase and NCX.

In pacemaker cells, the absence of a stabilizing I_K1_ is responsible for a more positive resting membrane potential (Cho et al., 2003). The non-specific cation current I_f_ (channel protein HCN) can thus generate diastolic depolarization leading to the generation of APs (Bers, 2002b).

Several mechanisms of arrhythmogenesis involving K channels have been described. Reduced function of Kv7.1 and hERG are the hallmark of congential long QT syndrome 1 and 2, respectively (Brenyo et al., 2012). A smaller I_Ks_ and I_Kr_ results in prolonged repolarization that is associated with torsade de pointes and sudden cardiac death (Roden, 2008). The underlying arrhythmic mechanisms involve increased triggered activity due to early afterdepolarizations (EADs) or reentry due to increased spatial heterogeneities in repolarization (see below). Recently, a mutation of an ATP-sensitive K channel (Medeiros-Domingo et al., 2010) has been identified in a patient with early repolarization syndrome, which is characterized by a prominent J wave on the ECG (see below) and is associated with an increased risk of ventricular fibrillation (VF) and cardiac death (Tikkanen et al., 2009). It was shown that this mutation results in gain of function in K_ATP_ (Medeiros-Domingo et al., 2010), consequently resulting in increased transmural heterogeneity of repolarization (see below).

Interestingly, besides rare congenital disease, altered K channel function has also been described for HF. It was shown that decreased I_K1_ and I_to_ density could lead to AP prolongation (Kaab et al., 1998).

Increased triggered activity is an important consequence of prolonged repolarization. The longer phase 2 of the AP results in reactivation of Ca channels that generate a depolarizing current possibly resulting in an EAD and ultimately leading to a triggered AP (Weiss et al., 2010). On the other hand, K channels have been also been shown to be involved in the generation of delayed afterdepolarizations (DADs) that are a consequence of cytosolic and sarcoplasmic reticulum (SR) Ca overload. The latter causes an increased propensity of spontaneous ryanodine-receptor (RyR) activation leading to a depolarizing inward NCX current (Kääb et al., 1996). Interestingly, this inward NCX current is more likely to induce DADs if I_K1_ is functionally downregulated, causing an unstable resting membrane potential (Dhamoon and Jalife, 2005).

Differential K channel expression across the ventricular wall is the basis for transmural dispersion of repolarization (TDR, Antzelevitch and Fish, 2001). Physiologically, the endocardial myocyte has a smaller I_to_ amplitude compared to the epicardial myocyte. This, together with increased depolarizing currents, contributes to a more positive AP plateau and a longer AP duration in the endocardial compared to the epicardial myocyte. The result is a physiological TDR that also determines the positive T wave on the surface ECG. However, under pathophysiological conditions this fine balanced regional difference in K channel function can be substantially altered. A preferential shortening of the epicardial AP by enhanced I_to_, for instance, together with a preferential prolongation of the endocardial AP by enhanced late I_Na_ and minor changes in the small I_to_ would increase the TDR. While a TDR increase in phase 1 and 2 of the AP results in the occurance of a J wave (positive deflection at the QRS-ST junction; Yan and Antzelevitch, 1996), increased TDR in phase 3 and 4 can cause abnormal T waves. If the increase in TDR in phase 3 and 4 reached a threshold, abnormal electrical activity would find excitable myocytes, resulting in reentry and leading to torsade de pointes (Yan and Antzelevitch, 1996). Computational modeling of a rabbit ventricular myocyte overexpressing CaMKII was used to investigate the importance of the expression level of I_to_ for AP duration (Grandi et al., 2007). If 100% I_to_ expression was used ( = epicardial myocytes), CaMKII overexpression resulted in a shortening of the AP duration mainly due to a CaMKII-dependent enhancement of I_to_. With 10% I_to_ expression ( = endocardial myocytes), however, AP duration increased because CaMKII-enhanced late I_Na_ and L-type Ca current outweighed the effect on the smaller I_to_. The mechanisms by which CaMKII alters potassium channel expression and function will be discussed in this review.

## CaMKII AND HF

Calcium-Calmodulin-dependent kinase II is a serine/threonine kinase that can regulate multiple ion channels and transporters including K channels (see below). Currently, four isoforms and up to 30 splice-variants of the serine/threonine CaMKII have been identified, with CaMKIIδ as the predominant cardiac isoform (Maier and Bers, 2007). CaMKII contains an N-terminal catalytic kinase-domain with an ATP-binding site as well as substrate binding sites. Adjacent to the catalytic subunit, an autoregulatory domain with a calmodulin (CaM)-binding site and important regulatory threonine (T287, T306, T307) and methionine residues (M281/282) precedes the C-terminal association-domain, which is critical for the assembly of the holoenzyme. *In vivo*, self-association of CaMKII holoenzymes forms two ring-like CaMKII-hexamers which are stacked on top of each other (dodecameric configuration; Rellos et al., 2010). CaMKII is activated by binding of a Ca/CaM complex to its autoregulatory domain, resulting in conformational changes which expose the catalytic subunit, enabling ATP and substrate binding. An important substrate is the autoregulatory domain of an adjacent subunit, resulting in inter-subunit phosphorylation at T287 (auto-phosphorylation). The latter enables CaM-independent activity after the dissociation of Ca/CaM (Maier and Bers, 2007). Novel alternative activation pathways have also been described involving oxidation or glycosylation at M281/282, both of which result in Ca-independent activity similar to auto-phosphorylation (Erickson et al., 2008, 2013).

CaMKII has been associated with HF development. In human HF, expression and activity of CaMKII is increased (Hoch et al., 1999; Kirchhefer et al., 1999; Ai et al., 2005). Moreover, CaMKIIδ-transgenic mice develop HF with increased AP duration, disturbed Ca handling, and are prone to ventricular arrhythmias (Maier, 2003; Wagner et al., 2011). In contrast, transgenic CaMKII inhibition or CaMKII knockout prevents cardiac remodeling and HF development after myocardial infarction or increased afterload (Zhang et al., 2005; Backs et al., 2009; Ling et al., 2009).

## TRANSIENT OUTWARD K CURRENT

**FIGURE 2:**
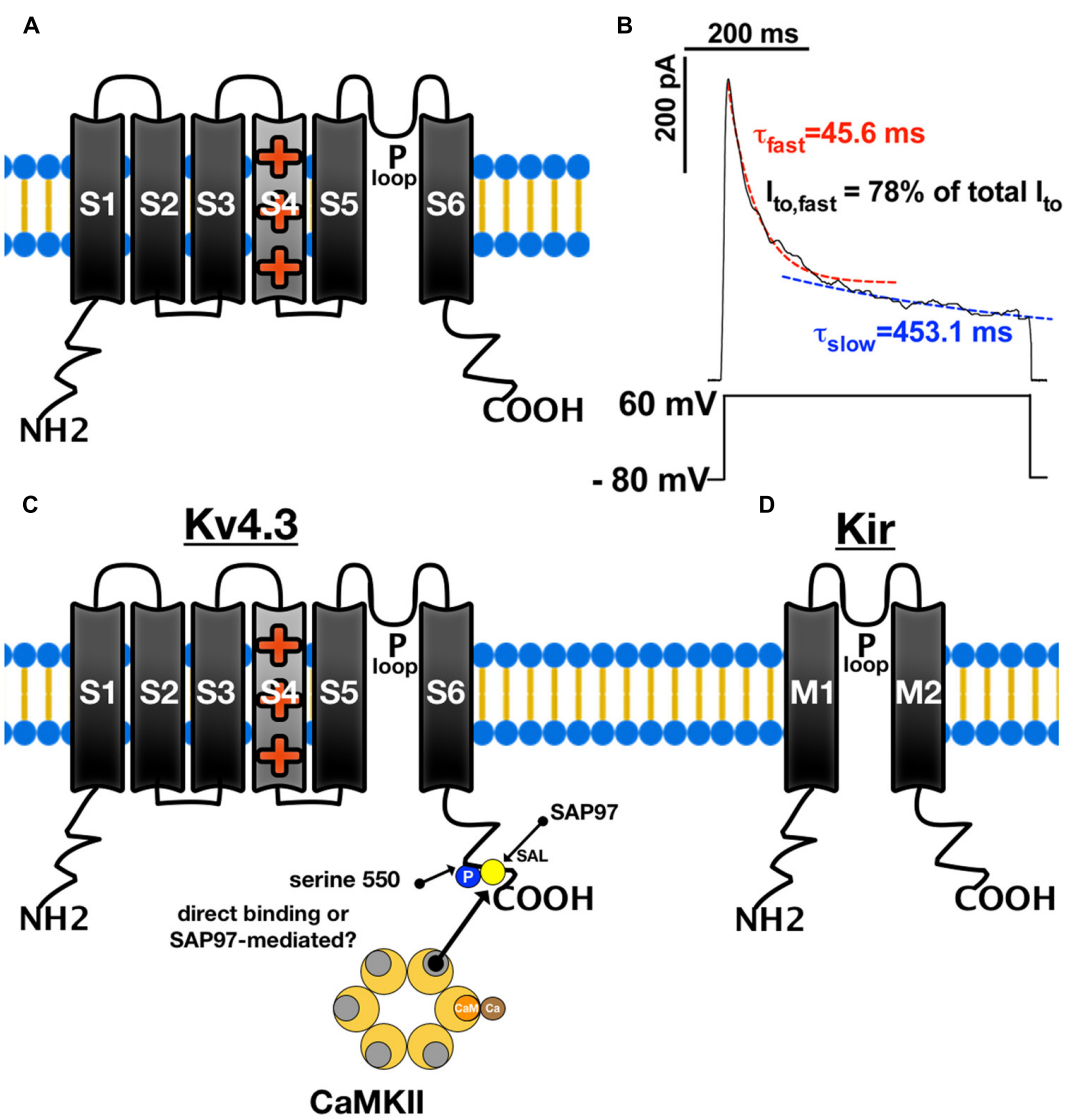
**Structure and function of K channels.** **(A)** Structure of voltage-gated K channel α-sunbunit (Kv) with six transmembrane segments (S1–S6). The S5–S6 segments face each other to form the central pore. The P-loop between the S5 and S6 segments acts as an ion conductance pathway and its signature motif G(Y/F)G functions as a K ion selectivity filter. Segment S4 senses voltage and moves outward during cell membrane depolarization resulting in conformational changes which open the pore. **(B)** There are two current components of I_to_ generated that can be distinguished according to their inactivation kinetics. I_to,fast_ inactivates with time constants (τ) of less than 100 ms, whereas the I_to,slow_ inactivates with τ of about 200 ms. **(C)** CaMKII can bind to Kv4.3 and phosphorylate serine 550 of its C terminus, which leads to altered current kinetics. SAP97 can also bind to Kv4.3 [at its Ser-Ala-Leu (SAL) segment] and possibly mediates the CaMKII-Kv4.3 interaction. **(D)** Inward rectifying potassium channels are formed by four α-subunits containing only two transmembrane segments (M1–M2) with a central P-loop as ion conductance pathway.

I_to_ is generated by a pore-forming α-subunit with six transmembrane segments (S1–S6). Accessory β-subunits can associate with this α-subunit (**Figure 2A**, Niwa and Nerbonne, 2010). In their either homologous or heterologous tetrameric assembly, the subunits’ S5 and S6 segments face each other to form the pore, while segment S4 senses voltage (Snyders, 1999; Niwa and Nerbonne, 2010). I_to_ is critical for the early repolarization (“notch”) immediately following the upstroke in phase 0 of the cardiac AP. There are at least two components of I_to_ generated by different channel isoforms, that can be distinguished according to their recovery and inactivation kinetics (**Figure 2B**; Brahmajothi et al., 1999). The fast component (I_to,fast_) inactivates and recovers with time constants (τ ) of less than 100 ms, whereas the slow component (I_to,slow_) inactivates with τ of about 200 ms and recovers with τ ranging from hundreds of milliseconds up to several seconds (Brahmajothi et al., 1999; Xu et al., 1999).

In human, rat, and canine tissue, I_to_ is generated mostly by the rapidly recovering channel population Kv4.3 (KCND3, Dixon et al., 1996). In a tachycardia-induced canine model of HF, a reduced ventricular Kv4.3 protein expression has been reported along with decreased I_to_ density (Zicha et al., 2004). It has also been shown that Kv4.3 expression is significantly reduced in human HF and that this is associated with a significant decrease in I_to_ density (Kaab et al., 1998; Zicha et al., 2004). Reduced I_to_ density is known to contribute to AP prolongation and prolonged QT intervals (Barry et al., 1998).

Despite this important role of Kv4.3 for I_to_ in human cardiac tissue, many animals species show a rather heterogeneous channel population comprised of Kv4.3, Kv1.4 (*KCNA4*), Kv4.2 (*KCND2*), and accessory KChIP subunits. In these species, I_to_ can be separated into the fast and slow component with varying relative contributions to total I_to_. In rabbit and mouse cardiac myocytes, for instance, Kv1.4 has been shown to be responsible for the slow component while a complex of Kv4.2, Kv4.3, and KChIPs is responsible for the fast component (Niwa and Nerbonne, 2010). Similar to the dog model of HF, tachycardia-induced HF in rabbits showed reduced total I_to_ density while AP duration was prolonged (Rose, 2005). Interestingly, mRNA levels of Kv1.4 and Kv4.2, as well as KChIP2, were significantly reduced, while Kv4.3 mRNA was unchanged. Protein expression of Kv4.2 and KChIP2 was significantly reduced, though Kv4.3 and Kv1.4 were unchanged (Rose, 2005). In a TNF-α-overexpressing mouse model of HF, I_to,fast_ density and Kv4.2 protein expression was significantly reduced (Petkova-Kirova, 2006). Other mouse models of HF exhibited similar reductions in I_to_ density and increased AP duration (Knollmann et al., 2000; Mitarai, 2000).

The differential regulation in the expression and function of the various channel isoforms underlying I_to_ suggests that the two components I_to,fast_ and I_to,slow_ are functionally and structurally independent ion currents.

## CaMKII-DEPENDENT REGULATION OF I_**to**_ EXPRESSION

CaMKII has been shown to influence the expression of channel isoforms underlying I_to_. In mice overexpressing CaMKIIδ, it was shown that total I_to_ density is significantly reduced (Wagner et al., 2009). This reduction was secondary to a reduced expression of Kv4.2 with reduced I_to,fast_ and accompanied by a prolongation of the cardiac AP (Wagner et al., 2009). In contrast, expression of Kv1.4 and I_to,slow_ were increased but this increase could not fully compensate the reduction in I_to,fast_ (Wagner et al., 2009). Interestingly, chronic CaMKII inhibition in mice by transgenic expression of the specific CaMKII inhibitory peptide AC3-I, a derivative of CaMKII substrate autocamtide-3, resulted in an increase in I_to,fast_ and shorter AP duration. On the other hand, the increase in I_to_ density was absent in crossbred mice expressing AC3-I but lacking phospholamban (PLN; Li et al., 2006). Since mice overexpressing CaMKIIδ also develop HF and chronic CaMKII inhibition may also affect the SR, it is not clear whether these changes are CaMKII-specific or secondary to remodeling or interference with other pathways. This is supported by the fact that short-term overexpression of CaMKIIδ in rabbit myocytes increases I_to_ (Wagner et al., 2009). Similary, in silico experiments with simulated CaMKII overexpression in rabbit myocytes also led to an increase in I_to_ along with faster I_to, slow_ recovery from inactivation (Grandi et al., 2007). Furthermore, Li et al. (2006) found no change in the expression of pore-forming subunits Kv4.2/Kv4.3 underlying increased I_to_ but only a downregulation of the accessory subunit KChIP2, suggesting that the regulation of I_to_ is complex, involving many interacting partners. In this respect it is not surprising that Kv4.3 and Kv4.2 form large macromolecular complexes with other proteins such as diaminopeptidyl transferase-like protein 6 (DPP6) and Eps15 homology domain-containing protein 4 (EHD4) (Marionneau et al., 2011). Recent evidence suggests that these proteins are important for endocytosis, vesicular recycling and trafficking (Cai et al., 2013). Perhaps more importantly, it has been shown that KChIP1 clamps two adjacent Kv4.3 α-subunits together via two contact interfaces that interact with the N-termini of Kv4.3 (Pioletti et al., 2006; Wang et al., 2006b). This stabilizes K_V_4.3 tetramers and also exerts an influence on current kinetics with current density being increased, inactivation slowed, and recovery from inactivation enhanced (Wang et al., 2006b). In addition, KChIP1 has been shown to be essential for proper Kv4 trafficking to the membrane (Cui et al., 2008).

More evidence that the downregulation of Kv4 in HF after CaMKII overexpression may be secondary and not directly mediated by CaMKII is derived from experiments investigating the interaction of the MAGUK (membrane-associated guanylate kinase) protein SAP97 with Kv4.

In neurons, the interaction of the C-terminal Ser-Ala-Leu (SAL)-sequence of Kv4.2 with SAP97 has been shown to be crucial for trafficking of Kv4.2 to the synaptic membrane (Gardoni et al., 2007). Interestingly, this trafficking has been shown to be enhanced by CaMKII phosphorylation of SAP97 at Serin-39 (Gardoni et al., 2007). Furthermore, it was shown in cardiac myocytes that Kv4.2/Kv4.3 channels form complexes with SAP97 and CaMKII (El-Haou et al., 2009). In the same publication, suppression of SAP97 in rat atrial myocytes via shRNA led to a decrease in I_to_, whereas SAP97 overexpression resulted in enhanced I_to_. Moreover, expression of Kv4.3 lacking the C-terminal SAL-sequence or SAP97 silencing via shRNA abolished the co-precipitation with CaMKII (El-Haou et al., 2009). Also, inhibition of CaMKII with autocamtide-2 related inhibitory peptide (AIP) resulted in reduced I_to_ and the inhibition was more pronounced after SAP97 overexpression (El-Haou et al., 2009). Interestingly, recent evidence suggests that SAP97 is downregulated in patients with dilated cardiomyopathy (Szuts et al., 2013).

## CaMKII-DEPENDENT REGULATION OF I_**to**_ GATING

The first evidence for a CaMKII-dependent regulation of cardiac potassium channel gating came from a study investigating human atrial myocytes (Tessier et al., 1999). The myocytes were isolated from donors with either chronic atrial dilation or chronic atrial fibrillation (AF). Patch–clamp experiments showed that inactivation of I_to_ was accelerated by CaMKII inhibition with either KN-93 or AIP (Tessier et al., 1999). Moreover, Tessier et al. (1999) also showed an increased expression level of CaMKII in the atrium of donors with chronic atrial dilation or chronic AF. More evidence for CaMKII regulating I_to_ came from experiments using transgenic mice overexpressing CaMKIIδ_c_ and also short-term CaMKIIδ_c_ overexpression in rabbit myocytes. It was shown that the recovery from inactivation of I_to,fast_ and I_to,slow_ was enhanced by CaMKII overexpression (Wagner et al., 2009). This enhancement could be blocked by acute CaMKII inhibition suggesting that this was not secondary to HF development (Wagner et al., 2009). The underlying mechanism of CaMKII-dependent regulation of I_to_ may involve direct CaMKII-dependent phosphorylation of Kv4.2 at serine 438/459, and of Kv1.4 at serine 123 (Roeper, 1997; Varga, 2004). Patch-clamp experiments in HEK-293 cells transfected with Kv4.3 showed that addition of autothiophosphorylated (pre-activated) CaMKII slowed I_to_ inactivation consistent with the results obtained by Tessier et al. (1999) and enhanced recovery from inactivation (Sergeant, 2005). Conversely, HEK cells treated with CaMKII-inhibitor KN-93 or CaMKII-inhibitory-peptide displayed significantly accelerated current inactivation and slowed recovery from inactivation (Sergeant, 2005). Moreover, if the C-terminal Kv4.3 mutant (serine 550 to alanine) was expressed, I_to_ inactivation was enhanced and I_to_ recovery was slowed (Sergeant, 2005). Neither addition of autothiophosphorylated CaMKII nor dialysis of CaMKII inhibitors could affect I_to_ recovery in HEK cells expressing this Kv4.3 S550A mutant (Sergeant, 2005), supporting the concept that the C-terminus of Kv4.3 is a hotspot for CaMKII-dependent association and regulation of I_to,fast_ (**Figure 2C**). Further evidence for a direct regulation of I_to,fast_ by CaMKII comes from studies in rat ventricular myocyte lysates showing that CaMKII co-immunoprecipitates with both Kv4.3 and Kv4.2 (Colinas, 2006), and inhibition of CaMKII with KN-93 resulted in a significant acceleration of I_to_ inactivation even through recovery from inactivation was unaffected (Colinas, 2006).

Interestingly, this CaMKII-dependent enhancement of I_to_ may also be important for reactive oxygen-species (ROS) induced arrhythmogenesis. ROS are known to oxidize and activate CaMKII (Erickson et al., 2008; Wagner et al., 2011) and ROS-induced arrhythmias are known to be CaMKII-dependent (Wagner et al., 2011). Recently, it was proposed that ROS-dependent activation of I_to_ favors EADs by facilitating I_Ca _reactivation (Zhao et al., 2012).

Thus, CaMKIIδ_c_ appears to regulate both channel expression and/or trafficking, but also acutely regulates channel gating properties. In both cases, acute regulation results in an enhancement of I_to_. In contrast to this, chronic CaMKII overexpression that leads to HF development results in a reduction of I_to_ but this appears to be a secondary effect.

## Kv4.3 AS AN IMPORTANT REGULATOR OF CaMKII ACTIVITY

While Kv4.3 is an important target for CaMKII, it may also influence CaMKII localization and activity. Recently, in HEK-293 cells transfected with Kv4.3 and His-tagged CaMKII, it was shown that Kv4.3 binds to CaM-dissociated CaMKII competitively at its CaM binding site (residues 301 and 307; Keskanokwong et al., 2010). This binding was independent from the auto-phosphorylation status of CaMKII, since both constitutively active (T-287D) or inactive (T-287A) CaMKII-mutants could also bind to Kv4.3 (Keskanokwong et al., 2010).

Since the CaMKII inhibitor KN93 also binds CaMKII at the CaM binding site (Sumi, 1991), it is conceivable that KN-93 disturbs the interaction of CaMKII and Kv4.3. Consistent with this idea, Keskanokwong et al. showed that co-purification of Kv4.3 and CaMKII is abolished upon addition of KN-93. Furthermore, it was shown that application of the Kv4.3 blocker 4-aminopyridine (4-AP) disturbes the co-purification of CaMKII and Kv4.3 in HEK-293 cells, while CaMKII auto-phosphorylation is increased (Keskanokwong et al., 2010). Similarly, increased CaMKII activity was found in guinea pig ventricular myocytes treated with 4-AP (Wang et al., 2006a). Moreover, 4-AP-induced blockade of Kv4.3 in HEK-293 cells has been shown to result in increased apoptosis and enhanced CaMKII-auto-phosphorylation, while the authors were able to prevent apoptosis by inhibition of CaMKII with KN-93 (Zhang et al., 2012).

This suggests that Kv4.3 may function as a reservoir for inactive CaMKII-units and exert an influence on CaMKII activation levels (**Figure 2C**). In accordance with this hypothesis, *in vivo* overexpression of Kv4.3 in mouse ventricular myocardium via multiple-site virus injection decreased the level of phosphorylated CaMKII, while CaMKII expression was not affected. CaMKII bound to Kv4.3 was also shown to be protected from activation by systolic Ca transients (Keskanokwong et al., 2010). The Kv4.3-CaMKII interaction may also be important for the regulation of other CaMKII target proteins. For instance, it was shown that blockade of Kv4.3 with 4-AP results in increased I_Ca_ that could be blocked by buffering cytosolic Ca with BAPTA or application of AIP (Wang et al., 2006a).

As previously mentioned, Kv4.3 is downregulated in HF (Kaab et al., 1998) while CaMKII is upregulated (Hoch et al., 1999). The CaMKII-Kv4.3 interaction may thus be severely altered in HF, contributing to higher CaMKII activity. In this context, the previously mentioned role for SAP97 in the regulation of Kv4.3 expression and Kv4.3-CaMKII interaction may be important. Downregulation of SAP97 in HF (Szuts et al., 2013) may underlie reduced Kv4.3 and may contribute to increased CaMKII activity.

## INWARDLY RECTIFYING CURRENT I_K1_

In contrast to the voltage-gated K channels, inwardly rectifying potassium channels [Kir2.1 (*KCNJ2*), Kir2.2 (*KCNJ12*), Kir2.3 (*KCNJ4*), and Kir2.4 (*KCNJ14*)] are formed by four α-subunits containing only two transmembrane segments (M1–M2) with a central P-loop but without a voltage-sensor (**Figure 2D**; Hibino et al., 2010). The main characteristic of this class of potassium channels is inward rectification, which features a strong potassium conductance during hyperpolarization, but a decrease in ion conductance upon depolarization due to blockade of the pore by Mg, Ca and cell membrane polyamines (Matsuda et al., 1987; Matsuda and Cruz, 1993; Hibino et al., 2010).

This peculiar inward rectifying property of the Kir2.x channels that generate I_K1_ renders these channels important stabilizers of the resting membrane potential by neutralizing resting influx of positive ions (Fauconnier et al., 2005). In addition, Kir2.x channels also contribute to late-phase (phase 4) repolarization (Dhamoon, 2004; Dhamoon and Jalife, 2005; Fauconnier et al., 2005).

In sinoatrial myocytes, the expression of channels forming I_K1_ is notably reduced, which allows for an unstable resting membrane potential that can be depolarized by I_f_, thus inducing diastolic depolarization (Bers, 2002b).

There is evidence that Kir2.x isoforms can assemble as homo- or heterotetrameres (Zobel et al., 2003). The functional characteristics of I_K1_ depend very much on the Kir isoforms that comprise I_K1_ (Panama et al., 2010), since rectification of current at depolarized membrane potentials ( > -30 mV) is complete for Kir2.1 and Kir2.2, but incomplete for Kir2.3 (Dhamoon, 2004). There is great variability in the expression of these isoforms between left and right ventricle (Warren et al., 2003) but also atrium and ventricle (Gaborit et al., 2007). Similar to channel subunits generating I_to_, expression of the Kir isoforms appears to be strongly species-dependent (Jost et al., 2013).

## Ca OR CaMKII-DEPENDENT REGULATION OF I_**K1**_

I_K1_ functional expression also seems to be regulated differently under pathophysiological conditions. It was shown that I_K1_ density is reduced in failing rat ventricular myocytes (Fauconnier et al., 2005). Interestingly, this reduction was attenuated in the presence of high EGTA (10 mmol/L) and abolished if intracellular Ca was buffered with BAPTA (20 mM; Fauconnier et al., 2005). Moreover, activation of RyR by application of ryanodine or FK506 led to a similar reduction of I_K1_ density in non-failing wild-type rat ventricular cells and this effect could be blocked by Ca-buffering with BAPTA (Fauconnier et al., 2005). Whether this reduction occurs via direct Ca-dependent blockade of I_K1_ via the mechanism described (Matsuda and Cruz, 1993) or mechanisms involving altered expression/trafficking of the underlying Kir isoforms is, however, completely unknown. Fauconnier et al. (2005) also suggested the involvement of protein kinase C (PKC), since the PKC inhibitor staurosporine antagonized the effect of ryanodine on I_K1_. PKC has been shown to phosphorylate Kir2.1 at serine 64 and threonine 353, leading to reduced I_K1_ in human atrial myocytes (Karle, 2002). On the other hand, opposite results have recently been shown in canine ventricular myocytes. Addition of 900 nmol/L Ca in the patch pipette significantly increased I_K1_ current compared to measurements with 160 nM Ca (Nagy et al., 2013). Therefore, the effect of Ca on I_K1_ may be species-dependent. Supporting evidence comes from intact field-stimulated (1 Hz) canine right ventricular papillary muscle. Increasing extracellular Ca from 2 to 4 mmol/L, resulted in increased Ca transient amplitude and significantly shortened AP duration. This Ca-dependent AP shortening could be prevented by inhibition of I_K1_ using BaCl_2_. Moreover, BaCl_2_ preferentially prolonged AP duration at 4 mM [Ca]_o_ vs. 2 mM [Ca]_o_. The authors conclude that Ca-dependent enhancement of I_K1__,_ at least in canine myocytes, may be an important contributor to repolarization reserve and an endogenous negative feedback mechanism inhibiting the generation of DADs due to high Ca levels (Nagy et al., 2013). Moreover, Nagy et al. (2013) also showed that CaMKII inhibition with KN-93 abolished the Ca-induced activation of I_K1_, suggesting that CaMKII is also involved in I_K1_ regulation. Supporting evidence for a CaMKII-dependent activation of I_K1_ also comes from rabbit ventricular myocytes. Acute overexpression of CaMKIIδ by adenovirus-mediated gene transfer resulted in a significant increase in I_K1_ density that could be blocked by addition of CaMKII-inhibitory peptide AIP to the pipette (Wagner et al., 2009). In the same, study, transgenic CaMKIIδ overexpression in mice that develop HF, however, resulted in a reduced I_K1_ density and reduced expression of Kir2.1 (Wagner et al., 2009). Thus, the discrepancy between the studies showing either increased or decreased I_K1_ may be due to species-differences but this remains speculative. In accordance, mouse ventricular myocytes with transgenic inhibition of CaMKII showed an increased I_K1_ and a shorter AP duration without a significant change in Kir2.1 and Kir2.2 expression levels (Li et al., 2006).

Aside from the species, it could also be relevant if the studied model results in HF. It was shown that SAP97 co-immunoprecipitates with Kir2.2 in rat hearts (Leonoudakis et al., 2004). Interestingly, in human dilated cardiomyopathy, the co-localization of SAP97 with Kir2.x was shown to be disturbed (Szuts et al., 2013). This suggests that a mechanism similar to the above mentioned Kv4.x-SAP97 interaction may be present. Therefore, further studies are greatly needed to clarify the importance of Ca and CaMKII for the regulation of I_K1_ in different animal models and in human disease (**Table 1**).

**Table 1:** Synopsis of studies investigating I_**K1**_ and arrhythmias.

Species	Model	I_K1_Current	Phenotype	Reference
Rat ventricular myocytes	Myocardial infarction	↓		Fauconnier et al. (2005)
Canine ventricular myocytes	High intracellular calcium	↑		Nagy et al. (2013)
Rabbit ventricular myocytes	Adenoviral CaMKII overexpression	↑		Wagner et al. (2009)
Mouse ventricular myocytes	Transgenic CaMKII overexpression	↓	Polymorphic and monomorphic VTs	Wagner et al. (2006, 2009)
Mouse ventricular myocytes	Transgenic CaMKII inhibition (AC3-I expression)	↑		Li et al. (2006)
Mouse ventricular myocytes	Acute CaMKII inhibition by AC3-I dialysis	→		Li et al. (2006)
Mouse ventricular myocytes	Kir2.1 knock-down	↓	Less ventricular arrhythmias	Piao et al. (2007)
Mouse ventricular myocytes	Kir2.1 overexpression	↑	More ventricular arrhythmias	Piao et al. (2007)
Kir2.1-overexpressing [ESC]-derived myocytes	Transplantation of ESC-derived myocytes into mouse ventricles after MI	↑	Less spontaneous VTs, less inducible VTs	Liao et al. (2013)
Rabbit ventricular myocytes	Tachycardia-induced HF			Rose (2005)
Mouse ventricular myocytes	Calsequestrin-overexpression-induced HF	↓	QRS and QTc prolongation	Knollmann et al. (2000)
Mouse ventricular myocytes	Gαq-overexpression-induced hypertrophy	↓		Mitarai (2000)
Mouse ventricular myocytes	Calcineurin overexpression	→		Petrashevskaya et al. (2002)
Mouse ventricular myocytes	Dominant-negative Kv4.2 expression - induced HF	↓		Wickenden et al. (1999)
Mouse ventricular myocytes	Dominant-negative Kv4.2 expression	↓	QRS and QTc prolongation	McLerie (2003)
Mouse ventricular myocytes	Kir2.1 overexpression	↑	More inducible, more stable VTs	Noujaim et al. (2006)
Mouse ventricular myocytes	Kir2.1 overexpression	↑	Bradycardia, AF, AV-Block, PVC, short QT	Li (2004)
Guinea pig ventricular myocytes	Kir2.1 overexpression	↑	QTc shortening	Miake et al. (2003)
Guinea pig ventricular myocytes	Dominant-negative Kir2.1 expression (downregulation)	↓	QTc prolongation	Miake et al. (2003)
Human ventricular myocytes	Dilated or ischemic cardiomyopathy	↓	APD prolongation	Beuckelmann et al. (1993)
Canine ventricular myocytes	Tachycardia-induced HF	↓	APD prolongation, QTc prolongation, more VTs	Kääb et al. (1996)Pak et al. (1997)
Monolayers of cultured neo-natal rat ventricular myocytes	Homogeneous Kir2.1 overexpression	↑	No reentry arrhythmias inducible	Sekar et al. (2009)
Monolayers of cultured neo-natal rat ventricular myocytes	Heterogeneous Kir2.1 overexpression	↑	Inducible reentry arrhythmias	Sekar et al. (2009)
Monolayers of cultured neo-natal rat ventricular myocytes	Homogeneous Kir2.1 suppression	↓	No reentry arrhythmias inducible	Sekar et al. (2009)
Monolayers of cultured neo-natal rat ventricular myocytes	Heterogeneous Kir2.1 suppression	↓	Inducible reentry arrhythmias	Sekar et al. (2009)
Canine atrial myocytes	Tachycardia-induced HF	→	Inducible atrial fibrillation (AF)	Li et al. (2002)

## I_**K1**_ AND ARRHYTHMIAS

I_K1_ is generally regarded as anti-arrhythmic by stabilizing resting membrane potential. In a canine model of tachycardia-induced HF, reduced I_K1_ has been shown to increase the propensity for sudden cardiac death and ventricular tachycardia (Kääb et al., 1996). Also, loss of function mutations in *KCNJ2* have been associated with long QT syndrome (LQT7), in which increased AP duration and increased propensity for arrhythmias can be observed (Tsuboi and Antzelevitch, 2006).

On the other hand, contrasting results have been shown for wild-type Kir2.1 overexpressing mice that have an increased propensity for ventricular arrhythmias (Noujaim et al., 2006; Piao et al., 2007) or AF (Li, 2004). Kir2.1 knock-down in mice was associated with longer AP duration and a reduced incidence of premature ventricular contractions before and after AV node ablation, reduced arrhythmias due to extracellular hypokalemia, and a reduced incidence of halothane-induced ventricular tachycardia (Piao et al., 2007).

This discrepancy may be solved by the fact that both increase or decrease of I_K1_ can be pro-arrhythmic if there is a substantial spatial heterogeneity in the functional expression profile (Sekar et al., 2009). In accordance with this, gain-of-function mutations in *KCNJ2* can result in short QT syndrome (SQT3), which is also pro-arrhythmogenic (Brenyo et al., 2012).

## DELAYED RECTIFYING K CHANNELS

The three channels Kv1.5 (*KCNA5*), hERG (*KCNH2*)*,* and Kv7.1**(*KCNQ1*) comprise the group of the delayed rectifying K channels. They generate I_Kur_ (ultra rapid), I_Kr_ (rapid), and I_Ks_ (slow), respectively. Together, they are important currents for phase 3 repolarization.

I_Kur_ is only present in atrial myocardium. In chronic human AF, it was shown that AP duration is reduced, possibly contributing to the arrhythmogenic mechanisms (Wettwer, 2004). Evidence for a role of Kv1.5 in AF came from a study investigating pharmacological Kv1.5 inhibition in a canine model of AF (Regan et al., 2007). They could show that AF terminates if Kv1.5 is inhibited. Furthermore, SAP97 was reported to co-immunoprecipitate with Kv1.5 (Murata et al., 2001) resulting in increased I_Kur_ (Godreau et al., 2002; Eldstrom et al., 2003).

Since SAP97 and CaMKII have been shown to interact (El-Haou et al., 2009), CaMKII expression is increased in AF (Tessier et al., 1999; Neef et al., 2010), and given the similarities between SAP97-dependent Kv4.3 and Kv1.5 regulation (Tessier et al., 1999; Godreau et al., 2002; El-Haou et al., 2009), it seems tempting to speculate that CaMKII could also regulate Kv1.5. Interestingly, in human atrial myocytes it was shown that CaMKIIδ is especially localized at intercalated disks, the region where Kv1.5 is also located (Tessier et al., 1999). Furthermore, Tessier et al. (1999) showed that selective inhibition of CaMKII with KN-93 or AIP reduced the amplitude of the sustained component of outward K current (I_sus_), whereas inhibition of phosphatases with okadaic acid increased I_sus_ (Tessier et al., 1999; Tessier, 2001). This I_sus_ is regarded as mainly generated by Kv1.5 (Fedida et al., 1993), suggesting that CaMKII, possibly by phosphorylation, regulates Kv1.5 (Tessier et al., 1999).

Besides I_Kur_, other K currents may also be involved in AF. I_K1_, for instance, has been shown to be upregulated in AF possibly contributing to shortening of AP duration (Dobrev and Ravens, 2003).

I_Ks_ is comprised of the pore-forming α-subunit Kv7.1, but also the auxiliary β-subunit KCNE1 (Ruscic et al., 2013). Loss-of-function mutations in *KCNQ1* are linked to an increase in AP duration associated with long QT-syndrome type I, whereas gain-of-function mutations in *KCNQ1* are associated with short QT-syndrome (SQT2) (Brenyo et al., 2012) and familial AF (Chen et al., 2003). Additionally, loss-of-function mutations in *KCNE1* have been associated with long QT-syndrome 5 (Splawski et al., 1997), which points out the important role of KCNE1 for the generation of I_Ks_. Indeed it has been shown that KCNE1 is important in slowing down the movement of the voltage-sensor S4 of Kv7.1 upon depolarization, thus explaining the slow activation kinetics of I_Ks_ (Ruscic et al., 2013). Similar mechanisms may very well regulate other voltage-gated channels and underlie their distinct activation kinetics.

Interestingly, co-immunoprecipitation experiments in yeast cells expressing wild-type Kv7.1 or mutated Kv7.1 with truncated α-helices showed that calmodulin can bind to the C-terminus of Kv7.1 (Shamgar, 2006). This IQ-motif appears to be a hot spot for mutations: yeast 2-hybrid experiments indicated that *KCNQ1* mutations A371T and S373P, which are associated with LQTS, lose their calmodulin-Kv7.1 interaction.

Moreover, agarose-pulldown assays in HEK-293 cells revealed that LQTS-associated Kv7.1 mutants W392R, S373P, and A371T bound significantly less calmodulin than wild-type Kv7.1 (Shamgar, 2006). This disturbed calmodulin- Kv7.1 interaction may be important for channel expression. Cell surface expression experiments with biotinylated channel proteins showed that mutants with impaired CaM-binding are significantly less expressed than wild-type Kv7.1 (Shamgar, 2006). Interestingly, overexpression of calmodulin in HEK-293 cells either expressing wild-type Kv7.1 or mutant S373P showed significant increases in Kv7.1 (5x) as well as S373P (100x) cell surface and protein expression, which highlights the important role of calmodulin for I_Ks_ assembly and cell surface expression (Shamgar, 2006).

In addition, the CaM- Kv7.1 interaction may also be relevant for the regulation of I_Ks_ gating. Patch-clamp experiments of inside-out membrane from *Xenopus* oocytes showed that application of calmodulin antagonist W7 significantly reduced current density of Kv7.1/KCNE1, while an increase in Ca significantly shifted voltage-dependence of channel activation toward more hyperpolarized membrane potentials (Shamgar, 2006).

Thus, the interaction of calmodulin and Kv7.1 appears to be critical for expression and function of I_Ks_, with the intriguing possibility that regulatory mechanisms could also involve some form of CaMKII interaction with calmodulin and Kv7.1 or KCNE1.

## ATP-SENSITIVE POTASSIUM CURRENT K_**ATP**_

The ATP-sensitive potassium current K_ATP_, comprising of Kir6.1 (*KCNJ8*) and Kir6.2 (*KCNJ11*) α-subunits, plays an important role in ischemic preconditioning (Li et al., 2007). K_ATP_ can be a substrate for CaMKII: in mice expressing CaMKII-inhibitory peptide AC3-I, an increased K_ATP_ current density has been shown along with an increase in the sarcolemmal Kir6.2 membrane surface expression (Li et al., 2007). Also, recent evidence from pancreatic β-cells suggests that Kir6.2 can be phosphorylated by CaMKII at threonine 224 (Kline et al., 2013). Co-expression of CaMKII and Kir6.2 in COS-cells resulted in a decreased K_ATP_ current.

The significance of K_ATP_ in HF and arrhythmogenesis is still largely unknown. There is evidence suggesting that K_ATP_-channel opening with cromakalim produces more stable ventricular arrhythmias (Quintanilla et al., 2013). In addition, Langendorff-perfused canine failing hearts with induced VF showed an increased rate of spontaneous VF termination, if K_ATP_ was blocked with glibenclamide (Taylor et al., 2012). Also, recently, a mutation in cardiac Kir6.1 that is associated with gain of function has been identified in a patient with early repolarization syndrome (see above; Medeiros-Domingo et al., 2010).

On the other hand, K_ATP_-blockade with glibenclamide in non-failing canine hearts with induced VF delayed the termination of VF (Taylor et al., 2012). Thus, the role of cardiac K_ATP_ and its regulation by CaMKII has yet to be evaluated.

## SUMMARY

While there is increasing evidence for an involvement of CaMKII in the regulation of K channels, many discrepancies are not yet understood. These discrepancies result from the great variability in the expression profile of K channels in different species and disease models. The greatest evidence so far exists for CaMKII-dependent regulation of Kv4.x expression, trafficking and function. Most intriguingly, the Kv4.x macromolecular complex appears to serve as a hotspot and reservoir for CaMKII, which may have profound impact on the regulation of various other CaMKII targets like Ca channels. CaMKII expression and activity has been shown to be increased in many animal models of HF, but also in human HF. Increased CaMKII activity has been shown to induce contractile dysfunction and arrhythmias. Therefore, a more detailed understanding of the mechanisms of K channel regulation by CaMKII is warranted.

## Conflict of Interest Statement

The authors declare that the research was conducted in the absence of any commercial or financial relationships that could be construed as a potential conflict of interest.
